# The association between appendicular skeletal muscle index and bone mineral density in children and adolescents with chronic kidney disease: A cross-sectional study

**DOI:** 10.1097/MD.0000000000036613

**Published:** 2023-12-15

**Authors:** Xuankai Qin, Jiahui Wei, Jinshuang Wei, Junyu Wei, Jie Chen, Fengying Lei, Yuanhan Qin

**Affiliations:** a Department of Pediatrics, The First Affiliated Hospital of Guangxi Medical University, Nanning, Guangxi, China.

**Keywords:** adolescents, appendicular skeletal muscle index, bone mineral density, chronic kidney disease, NHANES

## Abstract

Chronic kidney disease (CKD), a pervasive public health concern, can lead to complications like sarcopenia and reduced bone mineral density (BMD). However, it is still unclear exactly how muscle mass correlates with BMD in youngsters and adolescents with CKD. We aimed to investigate the association between appendicular skeletal muscle index (ASMI) and BMD among children and adolescents with CKD. In our research, we utilized data from the National Health and Nutrition Examination Survey (NHANES) collected between 2011 and 2014 to investigate the association of ASMI with BMD among this population. The association linking ASMI with total BMD was examined through multivariate linear regression models. Furthermore, fitted smoothing curves were employed, as well as generalized additive models. Our analysis finally included 503 CKD participants aged between 8 and 19 years. We found a significant association linking ASMI with total BMD among children and adolescents with CKD. The connection persisted even after accounting for covariates. Upon subgroup analysis, there was a statistically significant association of ASMI with total BMD for both males and females, as well as for Mexican-American and non-Hispanic White populations. However, no significant association was observed in other Hispanic, non-Hispanic Black, or populations of other races. We discovered a positive correlation linking the ASMI and the total BMD in children and teenagers with CKD. In CKD patients, maintaining skeletal muscle mass may be crucial for managing and preventing renal osteodystrophy.

## 1. Introduction

The worldwide incidence of chronic kidney disease (CKD) is on the rise, making it a significant public health concern.^[1]^ Sarcopenia, which involves a reduction of muscle mass and function, occurs frequently among CKD patients.^[2–4]^ Moreover, the correlation between sarcopenia and a higher likelihood of death has been noted in individuals with CKD.^[5,6]^

Fragility fractures are more likely to occur in CKD patients due to the frequent occurrence of reduced bone mineral density (BMD).^[7,8]^ Moreover, children with CKD face heightened risks of fractures and diminished bone mass, leading to a higher likelihood of developing skeletal fragility throughout life.^[9]^

Consequently, chronic kidney disease often leads to muscle wasting and renal osteodystrophy, which in turn leads to reduced physical mobility, heightened vulnerability, and an elevated likelihood of experiencing falls and fractures. Muscle and bone are closely related tissues that interact through complex hormonal, mechanical, and biochemical processes.^[10]^ The biomechanical interaction between bone and muscle can be disrupted by CKD.^[11]^ The development of the disease is associated with several mechanisms, including metabolic acidosis, lack of vitamin D, secondary hyperparathyroidism, and inflammation.^[12–14]^ However, despite understanding these mechanisms and prior studies showing a link between ASMI and BMD in adults, the association between muscle mass and BMD among young CKD patients is still unclear and poorly defined. A thorough understanding of the link between muscle mass and BMD can decrease fracture occurrences and enhance life quality for youngsters with CKD. A dependable measure of muscular tissue, appendicular skeletal muscle index (ASMI), can be utilized to make the diagnosis of sarcopenia.^[15]^ Hence, to address this gap, we used data provided by the National Health and Nutrition Examination Survey (NHANES), aimed to investigate the correlation between ASMI and total BMD within this population.

## 2. Materials and Methods

### 2.1. Study population

In this inquiry, we employed information from NHANES, a survey under the auspices of the National Center for Health Statistics (NCHS) gathering data on health and nutrition. As a program that assesses the health and nutrition of individuals of all ages within the US, NHANES serves as a valuable resource for researchers investigating various health conditions. In this research, we collected and analyzed information from 2011 to 2014, including information about population characteristics, results of laboratory tests, Dual-energy x-ray absorptiometry (DXA) scans, and measurements of the entire body.

**Figure 1.**
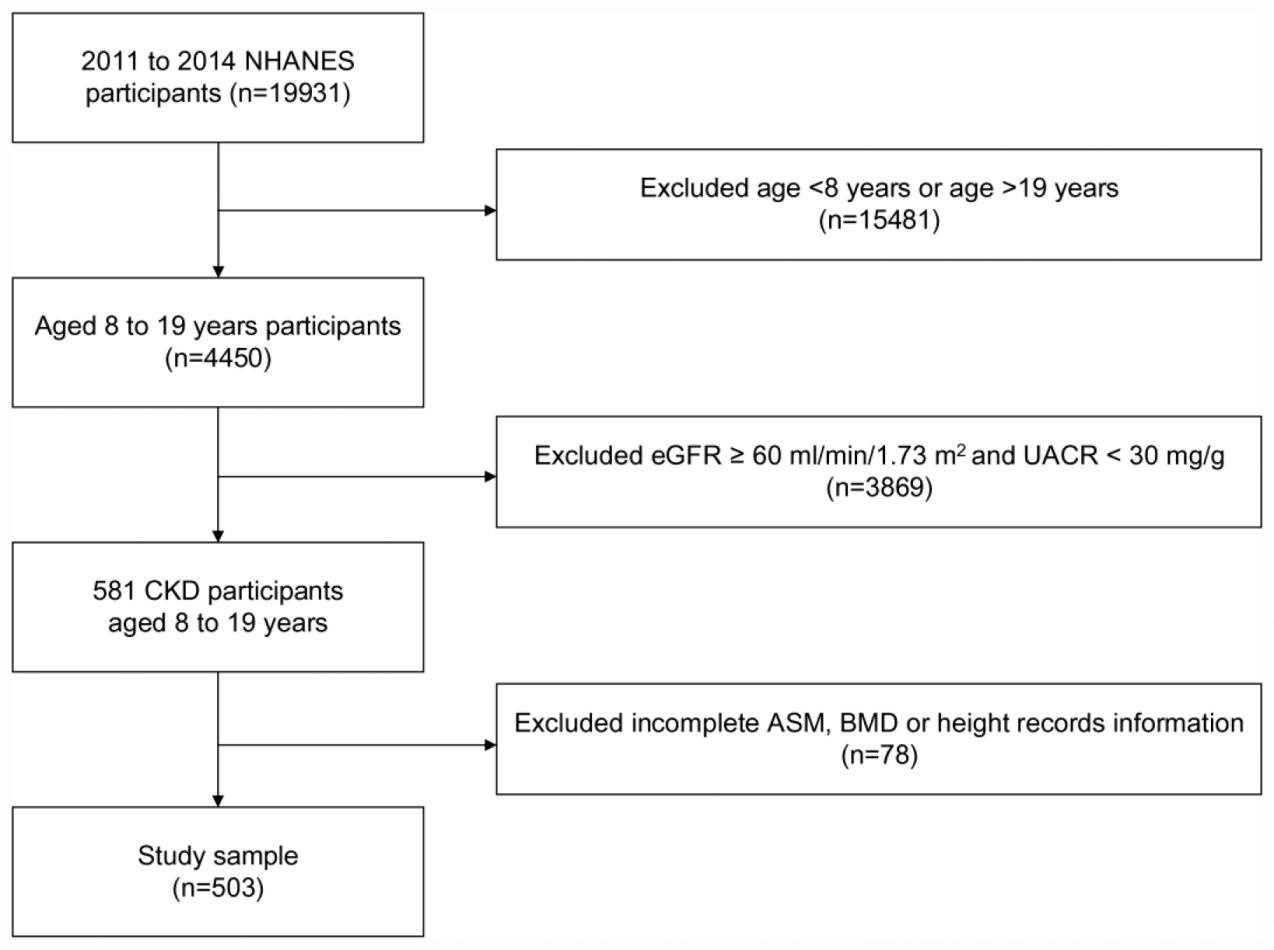
Flowchart of patient selection.

Our analyses were restricted to 4450 participants aged 8 to 19 years. Individuals with an estimated glomerular filtration rate (eGFR) of 60 mL/min/1.73 m^2^ or higher were not eligible for the study. Additionally, those with a urine albumin-to-creatinine ratio (UACR) of <30 mg/g were also excluded. After excluding subjects lacking information for ASM, total BMD, and height records, our final analysis included 503 participants (Fig. 1).

### 2.2. Chronic kidney disease

In our study, we employed the Chronic Kidney Disease Epidemiology Collaboration 2009 creatinine formula to determine the estimated glomerular filtration rate (eGFR) of individuals.^[16]^ An individual was considered to have CKD if their eGFR was below 60 mL/min per 1.73 m^2^ or if their urine albumin-to-creatinine ratio (UACR) was 30 mg/g or higher.^[17]^

### 2.3. Exposure and outcome variable

In this research, the exposure variable was represented by the appendicular skeletal muscle index. To accurately assess the amount of lean soft tissue present in the body, DXA scans were conducted on the entire body. These scans were obtained using equipment provided by Hologic, Inc., a company based in Bedford, Massachusetts. Mass of lean soft tissues in the arms and legs was added to calculate the appendicular skeletal muscle mass (ASM). Subsequently, the ASMI measure was computed through dividing ASM by the height squared, as indicated by the formula: ASMI = ASM/height^2^_._^[18]^

For this investigation, we employed total bone mineral density as the outcome variable. Qualified radiology technologists employed DXA to determine BMD. They employed Hologic Discovery model A densitometers and Apex software version 3.2 for this purpose.

### 2.4. Covariates

During our analysis and study, we accounted for various covariates that may have an impact on the association between ASMI and overall BMD to control for confounding bias. The factors considered encompassed various aspects like age, gender, ethnicity, body mass index (BMI), level of physical activity, alkaline phosphatase, calcium levels in the blood, phosphorus levels, potassium levels, albumin levels, creatinine levels, estimated glomerular filtration rate (eGFR), uric acid levels, as well as serum vitamin D. Our criteria for the inclusion of covariates encompassed factors that caused a 10% alteration in the effect value (β value) when variables were added or removed in the regression model, as well as factors that were derived from previous studies. In order to measure the levels of physical activity, we computed the number of weekly metabolic equivalent minutes (MET minutes) using the questionnaire about physical activity. For this research, individuals were grouped into low, moderate, high, and very high physical activity groups based on their adherence to national guidelines for physical activity. Low physical activity was defined as <500 MET-min/week, moderate as 500 to <1000 MET-min/week, high as 1000 to <1500 MET-min/week, and very high as 1500 or more MET-min/week.^[19]^ Serum samples were prepared, preserved, and transported to the lab for examination. The DxC800 system quantified ALP, calcium, phosphorus, potassium, creatinine, albumin, and uric acid in serum or plasma. The method of ultra-high performance liquid chromatography-tandem mass spectrometry was employed to quantify Vitamin D levels in serum.

### 2.5. Statistical analysis

We conducted univariate analysis to investigate the relationship between ASMI and the variables. To investigate the linear and nonlinear connections between ASMI and total BMD, we employed weighted multiple regression analysis in addition to smooth curve approximations. To validate the consistency of the connections among different groups, subgroup analysis was conducted. The mean ± standard deviation was used to express continuous variables that followed a normal distribution. On the other hand, for continuous variables that were not normally distributed, data were presented using the median and interquartile range. Categorical variables were represented by percentages. Statistical significance was determined by a *P* value that was below .05. R (version 4.1.3) and Empowerstats (accessible at https://www.empowerstats.net) were utilized as the main software tools for conducting all statistical analyses.

## 3. Results

### 3.1. Baseline characteristics of participants

Table 1 displays the attributes of the participants. This study was conducted on 503 CKD patients aged 8 to 19, whose average age was 12.96 years. There were 177 (35.2%) males and 326 (64.8%) females among the participants. Compared with male patients, females were less active and had significantly elevated BMI values, lower levels of ASM, ASMI, alkaline phosphatase, serum calcium, phosphorus, serum potassium, albumin, serum creatinine, serum uric acid, and serum vitamin D. However, no disparities in total BMD were observed between males and females.

**Table 1:** Characteristics of 503 patients with chronic kidney disease aged 8 to 19 yr.

	Male (n = 177)	Female (n = 326)	*P* value
Age (yr)	12.830 ± 2.984	13.035 ± 3.033	.457
Race (%)			.119
Mexican American	11.600	16.015	
Other Hispanic	7.799	8.347	
Non-Hispanic White	61.638	49.718	
Non-Hispanic Black	12.505	17.650	
Other Race	6.458	8.270	
Body mass index (kg/m^2^)	19.463 ± 5.071	22.033 ± 6.889	.001
ASM (kg)	17.263 ± 6.953	14.413 ± 4.621	<.001
ASMI (kg/m^2^)	6.488 ± 1.559	5.918 ± 1.334	<.001
Physical activity (%)			<.001
Low	18.454	76.755	
Moderate	48.842	10.657	
High	25.923	8.303	
Very high	6.781	4.285	
Alkaline phosphatase (U/L)	179 (114,285)	85 (61,127)	<.001
Serum calcium level (mg/dL)	9.685 ± 0.314	9.559 ± 0.284	.001
Phosphorus (mg/dL)	4.678 ± 0.745	4.313 ± 0.638	.001
Serum potassium (mmol/L)	4.057 ± 0.386	3.943 ± 0.352	.016
Albumin (g/L)	45.740 ± 3.051	44.047 ± 3.110	<.001
Serum creatinine (µmol/L)	69.032 ± 17.717	59.283 ± 18.869	<.001
Serum uric acid (µmol/L)	321.495 ± 66.866	259.308 ± 53.078	<.001
eGFR (ml/min/1.73m^2^)	137.436 ± 21.197	132.024 ± 22.338	.056
UACR (mg/g)	56.115 (39.512, 158.140)	67.415 (41.887,126.296)	.508
Total BMD (g/cm^2^)	0.935 ± 0.146	0.932 ± 0.141	.832
Serum vitamin D (nmol/L)	67.540 ± 18.550	60.175 ± 21.282	<.001
Blood urea nitrogen (mmol/L)	4.018 ± 1.181	3.842 ± 2.013	.431

ASM = appendicular skeletal muscle mass, ASMI = appendicular skeletal muscle index, BMD = bone mineral density, eGFR = estimated glomerular filtration rate, UACR = urine albumin-to-creatinine ratio;

### 3.2. Univariate analysis of ASMI and variables

Through univariate analysis, we examined the correlations between ASMI and different variables. As depicted in Table 2, there exists a positive relationship between ASMI and factors including age, BMI, creatinine, uric acid, and potassium. Conversely, a negative correlation is evident between ASMI and eGFR, phosphorus, albumin, vitamin D, and serum calcium.

**Table 2:** Relationship of ASMI with different variables.

Variables	β (95%CI)	*P* value
Age (yr)	0.266 (0.231, 0.301)	<.001
Race		
Mexican American	0	
Other Hispanic	0.068 (−0.483, 0.619)	.808
Non-Hispanic White	0.228 (−0.145, 0.601)	.231
Non-Hispanic Black	0.741 (0.282, 1.200)	.002
Other Race	−0.286 (−0.850, 0.279)	.322
Body mass index (kg/m^2^)	0.180 (0.168, 0.193)	<.001
Physical activity		
Low	0	
Moderate	0.303 (−0.157, 0.762)	.198
High	−0.015 (−0.571, 0.540)	.957
Very high	0.476 (−0.405, 1.357)	.291
Alkaline phosphatase (U/L)	−0.001 (−0.003, 0.000)	.118
Serum calcium level (mg/dL)	−0.844 (−1.435, −0.253)	.006
Phosphorus (mg/dL)	−0.515 (−0.762, −0.268)	<.001
Serum potassium (mmol/L)	0.792 (0.315, 1.269)	.001
Albumin (g/L)	−0.114 (−0.169, −0.060)	<.001
Serum creatinine (µmol/L)	0.023 (0.014, 0.032)	<.001
Serum uric acid (µmol/L)	0.009 (0.007, 0.012)	<.001
eGFR (ml/min/1.73 m^2^)	−0.011 (−0.019, −0.002)	.011
Serum vitamin D (nmol/L)	−0.008 (−0.015, −0.002)	.013
Sex		
Male	0	
Female	−0.569 (−0.826, −0.313)	<.001

### 3.3. Association between ASMI and total BMD

Table 3 presents the findings from the multivariate regression analysis. We used 3 different models to estimate the connection between ASMI and total BMD through weighted multivariable regression analysis. In Model 1, without any covariates adjustment, a positive association was observed to exist linking the ASMI with total BMD [0.068 (0.062, 0.074)]. Moreover, this favorable correlation remained unchanged even after accounting for confounding factors in both Model 2 [0.037 (0.031, 0.043)] and Model 3 [0.066 (0.048, 0.084)]. Based on the results of the fully adjusted model (Model 3), it was found that each unit gain in ASMI was correlated with a rise of 0.066 g/cm^2^ in total BMD, indicating a positive correlation between higher ASMI values and elevated levels of total BMD.

**Table 3:** Association between appendicular skeletal muscle index and total bone mineral density (g/cm^2^).

	Model 1 β (95%CI) *P* value	Model 2 β (95%CI) *P* value	Model 3 β (95%CI) *P* value
ASMI	0.068 (0.062, 0.074) <.001	0.037 (0.031, 0.043) <.001	0.066 (0.048, 0.084) <.001
Stratified by gender			
Male	0.070 (0.061, 0.080) <.001	0.036 (0.027, 0.046) <.001	0.053 (0.021, 0.085) <.001
Female	0.070 (0.062, 0.079) <.001	0.038 (0.030, 0.046) <.001	0.089 (0.057, 0.121) <.001
Stratified by race			
Mexican American	0.076 (0.064, 0.088) <.001	0.046 (0.031, 0.060) <.001	0.082 (0.016 0.148) .022
Other Hispanic	0.079 (0.057, 0.101) <.001	0.048 (0.027, 0.069) <.001	−0.037 (−0.212, 0.138) .707
Non-Hispanic White	0.069 (0.055, 0.082) <.001	0.038 (0.025, 0.051) <.001	0.072 (0.036, 0.109) <.001
Non-Hispanic Black	0.051 (0.041, 0.060) <.001	0.029 (0.021, 0.038) <.001	0.018 (−0.022, 0.059) .387
Other Race	0.095 (0.075, 0.115) <.001	0.047 (0.032, 0.063) <.001	0.141 (−0.082, 0.364) .304

ASMI = appendicular skeletal muscle index

Model 1: No covariates were adjusted.

Model 2: Age, gender, and race were adjusted.

Model 3: Age, gender, race, body mass index, physical activity, alkaline phosphatase, serum calcium level, phosphorus, serum potassium, albumin, serum creatinine, estimated glomerular filtration rate, serum uric acid, and serum vitamin D were adjusted.

In the subgroup analysis stratified by gender or race, the model is not adjusted for the stratification variable itself.

Inconsistent connections between ASMI and total BMD were observed across subgroups. A significant correlation was observed between ASMI and total BMD within every gender-stratified subgroup. Regarding the subgroup categorized by race, the strong association between ASMI and total BMD remained significant among Mexican Americans and non-Hispanic Whites, while it was not observed in other Hispanics, non-Hispanic Blacks, or populations of other races.

**Figure 2.**
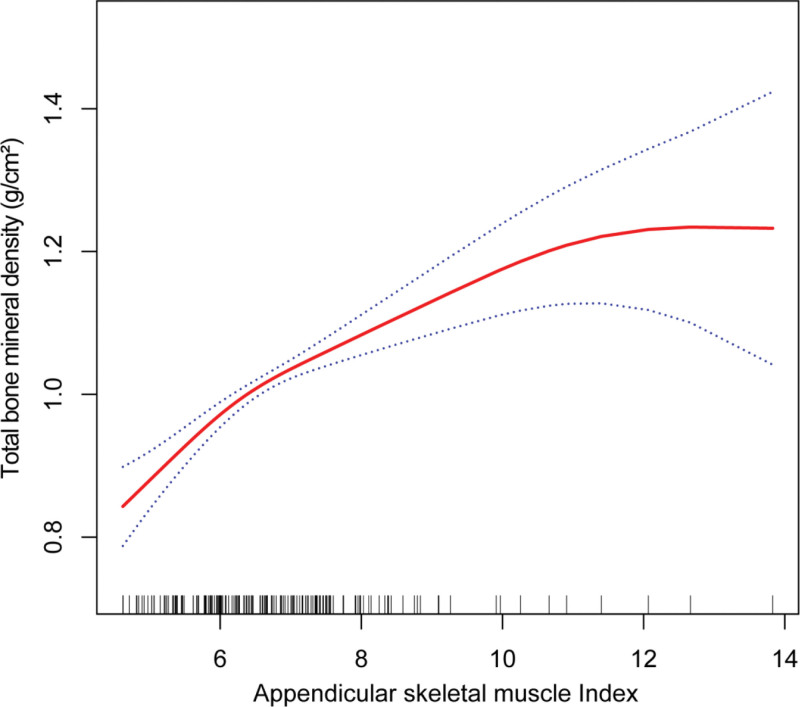
The association between appendicular skeletal muscle index and total bone mineral density. The solid red line represents the smooth curve fit between variables. Blue bands represent the 95% of confidence interval from the fit. Age, gender, race, body mass index, physical activity, alkaline phosphatase, serum calcium level, phosphorus, serum potassium, albumin, serum creatinine, estimated glomerular filtration rate, serum uric acid, and serum vitamin D were adjusted.

**Figure 3.**
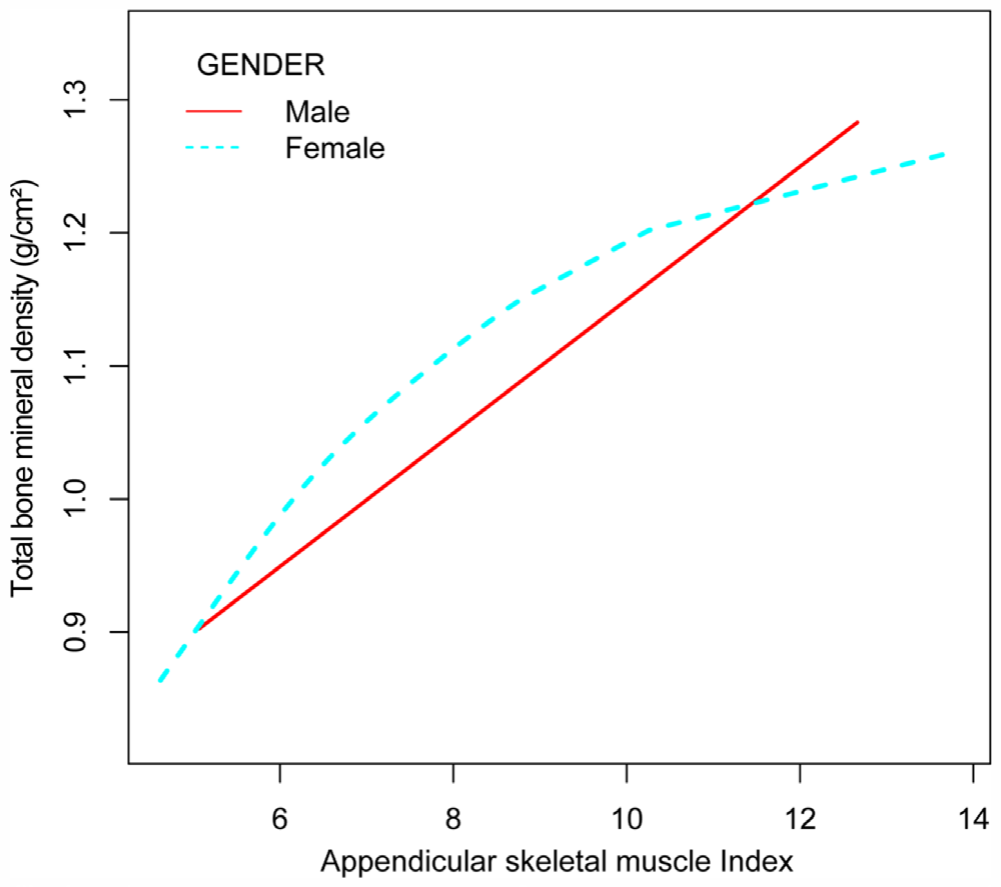
The association between appendicular skeletal muscle index and total bone mineral density, stratified by gender. Age, race, body mass index, physical activity, alkaline phosphatase, serum calcium level, phosphorus, serum potassium, albumin, serum creatinine, estimated glomerular filtration rate, serum uric acid, and serum vitamin D were adjusted.

**Figure 4.**
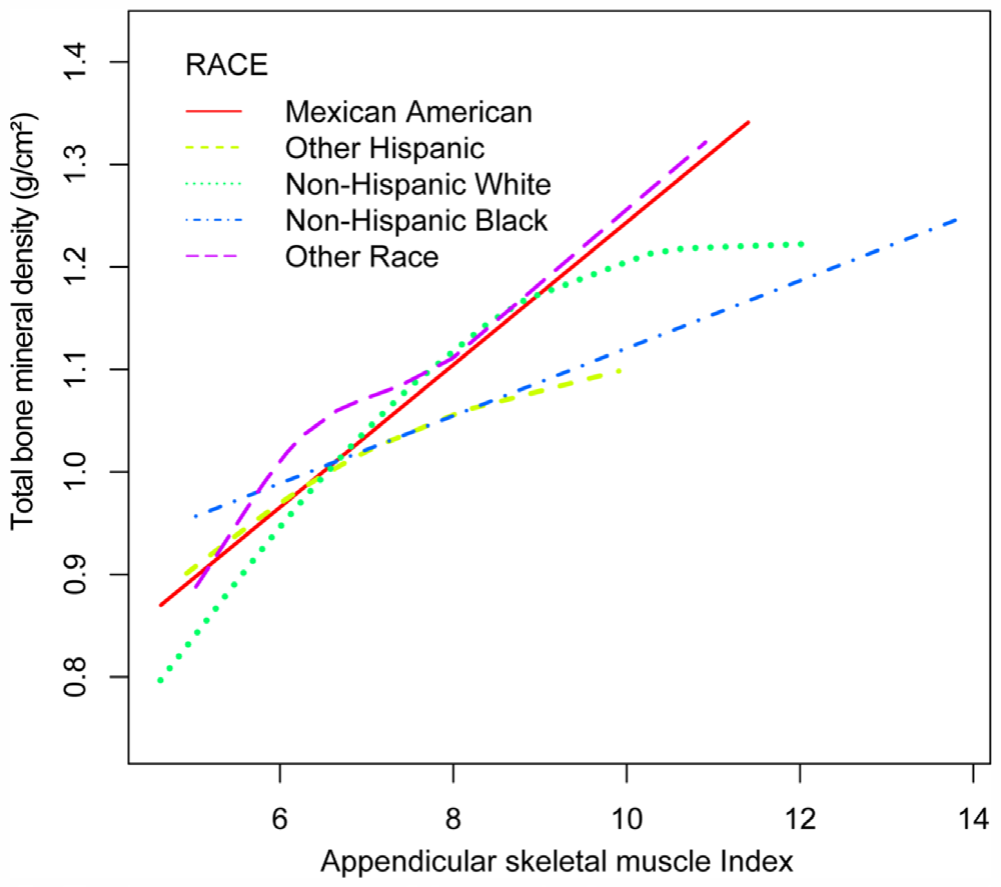
The association between appendicular skeletal muscle index and total bone mineral density, stratified by race. Age, gender, body mass index, physical activity, alkaline phosphatase, serum calcium level, phosphorus, serum potassium, albumin, serum creatinine, estimated glomerular filtration rate, serum uric acid, and serum vitamin D were adjusted.

Furthermore, we not only explored the linear correlation between ASMI and total BMD, but also explored the possibility of a non-linear relationship between the 2, as depicted in Figures 2–4.

## 4. Discussion

In this study representing the entire nation, we discovered a favorable correlation between ASMI and total BMD among children and teenagers with CKD. With each unit increase in ASMI, total BMD rises by 0.066 g/cm². Nonetheless, in the analysis of racial subgroups, the relationship between ASMI and BMD varied. It was significant among Mexican Americans and non-Hispanic Whites, but not among other Hispanics, non-Hispanic Blacks, or other racial populations.

At present, studies exploring the relationship between ASMI and BMD in children and teenagers are scarce. Most research examining this connection has been focused on adults, and differing associations have been observed. Multiple studies, which were conducted using a cross-sectional design, have shown a significant link between higher ASMI and greater BMD, indicating that individuals with increased muscle mass tend to possess higher bone density. For example, a study that included 948 participants between the ages of 40 and 59 discovered a favorable connection between ASMI and BMD in the lumbar spine.^[20]^ In another study, Julia et al, specifically targeted adults who have non-dialysis dependent chronic kidney disease, and discovered a correlation between decreased BMD and low muscle mass among this population.^[21]^ Additionally, a cross-sectional study examined males diagnosed with diabetes and revealed a favorable correlation between ASMI and both femoral neck BMD and hip BMD.^[22]^ Nevertheless, it is crucial to note that some studies have shown inconsistent findings. For instance, a cross-sectional observational study conducted in Brazil found no association between ASMI and BMD among postmenopausal women.^[23]^ Despite the fact that most research examining the association between ASMI and BMD is limited to adults, there are several clinical studies involving healthy adolescents that have identified a positive association between grip strength, a marker for muscle mass, and BMD. In a cross-sectional study of 118 adolescents aged 10 to 14 years, a positive relationship was found between grip strength and bone density.^[24]^ Moreover, a prospective study carried out in Brazil demonstrated a significant positive correlation between grip strength in childhood and BMD in adulthood.^[25]^

The exact mechanism of this positive association remains unclear. Potential elements that may contribute to this association encompass metabolic acidosis, insufficiency of vitamin D, secondary hyperparathyroidism, as well as inflammation. Muscle tissue, as well as bone tissue, both derive from the same mesenchymal precursor and interact through biochemical and mechanical pathways.^[26]^ CKD can disrupt this biomechanical crosstalk by affecting the cellular activity of both tissues. Adynamic bone disease can be caused by metabolic acidosis, which can impact calcium balance and synthesis of vitamin D.^[27]^ Earlier research discovered that metabolic acidosis has an impact on muscle as well, causing protein breakdown and disrupting the signaling of insulin/IGF-1 (Insulin-like Growth Factor 1), which ultimately results in a reduction of muscle strength.^[28,29]^ The well-being of bones and muscles in individuals with CKD can be impacted by vitamin D insufficiency, as well as the presence of secondary hyperparathyroidism. Due to low vitamin D levels, mineral deficiencies, as well as secondary hyperparathyroidism, can affect bone metabolism and increase fracture rates.^[12]^ In addition, the structure and metabolism of skeletal muscle can be affected by Vitamin D and secondary hyperparathyroidism.^[11]^ Inflammation could potentially serve as a fundamental factor since CKD is characterized by a widespread inflammatory condition that hinders the creation of bones and promotes bone breakdown. Multiple research studies have indicated that inflammation has the potential to modify the creation and breakdown of bones by means such as increasing the levels of RANKL (receptor activator of nuclear factor-B ligand) in osteoblasts while diminishing the function of Wnt/β-catenin pathway.^[14]^ Furthermore, the presence of pro-inflammatory cytokines can lead to muscle protein degradation.^[30]^

To the best of our knowledge, this research is the initial examination of the correlation between ASMI and BMD in youngsters and teenagers with CKD. One advantage of our research is that we utilized survey data collected from a wide range of individuals, incorporating sample weights to enhance the accuracy and inclusiveness of our results. Besides, we conducted further subgroup analysis to gain a deeper understanding. However, it is crucial to recognize the constraints of our research. Determining the causal relationship is challenging because of the cross-sectional design employed in our investigation. In order to fully comprehend the specific mechanism linking ASMI and total BMD among children and adolescents who have CKD, further research and large-scale prospective studies are necessary. Furthermore, it should be noted that some unmeasured confounding variables (such as parathyroid hormone levels and calcium supplements use) were not available for data collection in this study, which may have influenced our findings.

## 5. Conclusions

To sum up, this research showed a positive connection between ASMI and total BMD among children and adolescents who have CKD. Preserving skeletal muscle mass might be important to prevent and manage renal osteodystrophy in CKD patients. Nevertheless, additional research is required to validate or oppose our findings.

## Acknowledgments

We are grateful to Jing Zhang (Shanghai Tongren Hospital) for his contributions to the nhanesR package, whose nhanesR package and webpage have greatly facilitated our ability to extract data from the NHANES database.

## Author contributions

**Data curation:** Jiahui Wei, Jie Chen.

**Formal analysis:** Xuankai Qin.

**Funding acquisition:** Yuanhan Qin.

**Software:** Fengying Lei.

**Writing – original draft:** Xuankai Qin.

**Writing – review & editing:** Yuanhan Qin, Jinshuang Wei, Junyu Wei.
